# The Employment of Hydrogen Peroxide in Water Disinfection Poses a Threat to Aquatic Ecosystems Because of Its Toxicity to Nontarget Organisms

**DOI:** 10.1002/tox.70050

**Published:** 2026-02-11

**Authors:** Letícia Queiroz Almeida, Thayrine Dias Carlos, Leydiane Barbosa Bezerra, Rone S. Barbosa, Nelson Luís Gonçalves Dias Souza, Douglas Henrique Pereira, Renato Almeida Sarmento, Grasiele Soares Cavallini

**Affiliations:** ^1^ Programa de Pós‐Graduação em Produção Vegetal Universidade Federal do Tocantins Gurupi Tocantins Brazil; ^2^ Programa de Pós‐Graduação em Química Universidade Federal do Tocantins Gurupi Tocantins Brazil

**Keywords:** bioindicators, hydroxyl radicals, oxidation, planarians, water quality

## Abstract

Hydrogen peroxide is widely used in water and wastewater treatment, particularly in advanced oxidation processes that aid in the degradation of compounds and microbial disinfection. Despite concerns about potential environmental contamination, its impact on freshwater ecosystems remains inadequately studied. Evaluating the ecotoxicity of aquatic organisms is crucial for establishing safe environmental concentrations. This study focuses on assessing the impact of hydrogen peroxide on the freshwater planarian *Girardia tigrina* , a regenerative invertebrate serving as an ecotoxicological model. Both acute and chronic effects were evaluated, including locomotion, regeneration, and reproduction. The LC_50_ for 48 h was determined to be 123.55 mg/L. Sublethal exposure had minimal effects on locomotion, blastema regeneration, and fertility. Regeneration delays were observed at 3 mg/L, while fecundity was significantly affected at 1.52 mg/L. The planarians exhibited morphological abnormalities at the maximum quantity tested in the reproduction experiment (12.86 mg/L), which may have been the result of hydrogen peroxide‐induced mutations. In order to protect ecological health, this study advises keeping hydrogen peroxide levels below 1.52 mg/L and draws attention to the potential risks it poses to aquatic habitats. To guarantee complete environmental protection and a better knowledge of its effects, further research is needed.

## 1 Introduction

The quality of life in contemporary civilizations is significantly influenced by the safety of drinking water. Problems with microbial infections that arise during the production and distribution of drinking water can have a significant effect on public health [1].

Disinfection procedures aim to eliminate or render inactive pathogenic organisms, undesirable organisms, or other organisms to produce high‐quality water and prevent illness [2, 3]. Hydrogen peroxide is an efficient and versatile oxidant (1.77 V vs. SHE) and represents an appealing option when compared to chlorine dioxide and potassium permanganate due to its rapid decomposition, mainly into oxygen and water, resulting in minimal residues. The practice of treating water, sewage, and industrial effluents with hydrogen peroxide has been common for at least 20–25 years in developed countries [4].

Hydrogen peroxide is recognized as an efficient bactericide and algaecide and has advantages such as lower cost compared to chlorinated biocides and simpler and safer application when compared to chlorine [5]. Wang et al. [6] described that the residual concentration of 1.3–5 mg/L of hydrogen peroxide can affect biological processes, representing a critical impact on microbial ecology, and warned about the importance of establishing a maximum allowable concentration.

Ecotoxicological assessments are essential for determining the toxicity of oxidants and their immediate and long‐term effects on ecosystems, as a variety of biotic and abiotic stress factors naturally affect the aquatic ecosystem, which can result in alterations to the physical and chemical composition of the environment [7, 8].

Since the early 1970s, when specialists in the field became increasingly involved in the conservation of freshwater systems, the use of aquatic organisms as indicators of the quality of aquatic environments has been widely adopted. This trend has culminated in public pressure on government authorities to restore these ecosystems through biomonitoring programs [9].

The use of invertebrate bioindicators has received increased attention in ecotoxicological studies due to their relatively high sensitivity to environmental alterations. For this reason, their reactions to environmental changes provide information on population and community dynamics, establishing them as promising screening instruments for predicting the acute and chronic toxicities of pollutants [10, 11].

Among freshwater bioindicators, planarians are especially recommended as test organisms for assessing the genotoxicity of substances and environmental changes because of their regenerative capacity and high sensitivity to environmental pollution [12]. Their remarkable ability to regenerate lost body parts allows these free‐living organisms to be used in studies aimed at morphological and functional restoration, especially by regulating the proliferation of neoblasts (stem cells) in response to changes in the metabolic state and injuries [13]. Planarians are important platyhelminths in freshwater habitats worldwide, both in terms of species richness and abundance, and are predators of small crustaceans and larvae [4].

Studies with different disinfectants in aquatic environments, using planarians as bioindicators, reinforce the importance of these organisms in environmentally relevant ecotoxicological responses. In acute tests, the results of a study with the planarian *Dugesia tigrina* showed that the active chlorine of calcium hypochlorite presented greater toxicity (LC_50_ = 2.63 mg/L), while peracetic acid showed higher LC_50_ values (3.16 mg/L), indicating lower toxicity [4].

This study stands out for providing the first integrated assessment of the acute and sublethal effects of H_2_O_2_ on *Girardia tigrina* , including LC_50_ determination, regeneration analyses, reproduction, morphology, and identification of environmentally relevant sublethal concentrations, highlighting the importance of bioindicators in monitoring and preserving the environmental quality of aquatic ecosystems.

## 2 Materials and Methods

### 2.1 Physicochemical and Microbiological Characterization of Water

The water used for the disinfection test came from the Água Franca River, Gurupi, Tocantins, Brazil. The water sample was characterized by the following parameters: turbidity, pH, Abs_254nm_, *Escherichia coli* and total coliforms [14, 15]. For microbiological analyses, the membrane filtration technique was used, in which 100 mL of the sample was filtered through a sterile cellulose nitrate membrane with a porosity of 0.45 μm, which was placed in Petri dishes containing the selective and differential chromogenic culture medium for coliforms and incubated at 36°C for 24 h [16].

### 2.2 Description and Specifications of Materials

The assay was performed using Dinâmica brand P.A.—ACS hydrogen peroxide. The peroxide concentration analyses were performed using the Vacu‐vials Peroxide CHEMetrics Kit spectrophotometric method by ferric thiocyanate at 270 nm.

### 2.3 Cultivation and Maintenance of *G. tigrina* Planarians

The *G. tigrina* planarians used in this study were originally obtained from the University of São Paulo and have since been maintained and cultivated at the Laboratory of Applied and Functional Ecology of the Federal University of Tocantins, Gurupi, Tocantins, Brazil. The organisms were kept in standardized ASTM medium [17], ensuring controlled environmental conditions. The culture was maintained in plastic containers kept in the dark, with the temperature controlled at 22°C ± 1°C, the pH adjusted to 7.5, and continuous aeration. Feeding was performed weekly using bovine liver, provided ad libitum for a period of 5 h. After this interval, the planarians were transferred to clean containers, and the medium was completely renewed [18, 19, 20].

### 2.4 Ecotoxicological Assays

Ecotoxicological assays were performed according to the culture and evaluation protocols used by the research group [4, 12, 21, 22]. For the bioassays with *G. tigrina* , the lethal concentration (LC_50_) and sublethal effects (locomotion, regeneration, and reproduction) of hydrogen peroxide were determined.

In the acute test, organisms measuring 8 mm (±0.1 mm) were selected and underwent a fasting period of 1 week before the experiments began. To determine the LC_50_, experimental solutions of hydrogen peroxide were prepared by dilution in ASTM medium, testing concentrations of 16, 50, 83, 25, 116, and 166.5 mg/L, with reconstituted medium as a control. The hydrogen peroxide concentration was analyzed using the Vacu‐vials CHEMetrics kit, followed by spectrophotometric readings on the PG Instruments T60 UV/VIS spectrophotometer.

The physicochemical parameters were also measured. The experiment was conducted in Petri dishes (*Ø* = 90 mm) containing 20 mL of the concentrations, using five organisms per dish and replicating each treatment five times. The mortality of *G. tigrina* was evaluated after 24, 48, and 96 h of exposure in a static system at 22°C ± 1°C. Planarians displaying immobility in response to light or exhibiting total or partial body degeneration were considered dead.

For the chronic locomotion and regeneration test, planarians measuring 8 mm (±0.1 cm) in length were exposed for 8 days to four nominal treatment concentrations of hydrogen peroxide, 1.52, 3, 6.01, and 12.86 mg/L, defined as 10% of the LC_50_ value found in the acute test, in addition to the ASTM medium control. The hydrogen peroxide concentration was analyzed using the Vacu‐vials CHEMetrics kit, followed by spectrophotometric readings on the PG Instruments T60 UV/VIS spectrophotometer. Each treatment was replicated three times, with each replicate containing 12 planarians. The exposure took place in 200 mL glass vials, each containing 100 mL of the experimental solution. The entire process was conducted in a controlled environment at a temperature of 22°C ± 1°C, using a static system and maintaining darkness throughout the exposure period.

### 2.5 Locomotion

Following an eight‐day exposure to hydrogen peroxide, quantification of the parameter of locomotion speed (pLMV) in *G. tigrina* . To carry out this assessment, we placed each planarian individually in an aluminum container (Ø = 75 cm), with millimeter paper lining the bottom and an adequate amount of ASTM medium to encourage movement. The planarians were positioned at the center of the millimeter paper, allowing them to move freely across its surface. Each line on the millimeter paper represented 0.5 cm. The calculation of the pLMV was performed after a 3‐min observation period to determine their locomotion speed.

### 2.6 Regeneration

Within each treatment group, the subjects were exposed to 12 planarians to decapitation immediately following the exposure period, with a single cut made behind the auricles using a sterilized scalpel blade. Subsequently, the decapitated planarians were relocated to Petri dishes filled with 20 mL of ASTM medium. Initially, assessments were conducted at 24‐h intervals for 2 days, focusing on the measurement of blastema length in millimeters. Following this initial period, we monitored the development of photoreceptors and auricles every 12 h, using a stereoscopic microscope for observation.

### 2.7 Reproduction

Adult planarians of early reproductive age, measuring 1.4 cm (±0.2 cm) in total length, were selected and exposed to hydrogen peroxide for 4 weeks. The experimental design consisted of four replicates, each with 10 organisms, all placed in glass vials containing 100 mL of the experimental solution. Weekly, the medium was refreshed following their feeding with bovine liver. These planarians were maintained in a light‐controlled environment at a consistent temperature of 22°C ± 1°C. During exposure, to assess reproductive activity, quantification was performed of the number of cocoons and flatworms, according to the methodology described by Knakievicz et al. [23]. The fecundity rate (Fc) was determined by dividing the number of cocoons produced per week by the total number of planarians exposed, while the fertility rate (Fr) was determined by the number of planarians produced per individual or pair per week.

### 2.8 Statistical Analyses

The data were analyzed using GraphPad Prism software version 7.0 for Windows (GraphPad Software La Jolla, California, USA). Our analysis included subjecting all the data to ANOVA, preceded by the Shapiro–Wilk test to assess normality and Bartlett's test to assess equality of variances. Notably, the data relating to the impact of hydrogen peroxide on the formation of photoreceptors and auricles did not meet the assumption of equality of variance. Consequently, we opted for a non‐parametric analysis method, specifically the Kruskal–Wallis test, followed by Dunn's post hoc test for multiple comparisons.

## 3 Results

### 3.1 Disinfection Test With Hydrogen Peroxide

The physicochemical and microbiological characterization of the water sample, in different parameters, is presented in Table 1.

**TABLE 1:** Physicochemical and microbiological characterization of river water.

Parameter	River water
pH	6.8
Turbidity	12 NTU
Abs_254nm_	0.071
*E. coli*	*N*_0_ = 120 CFU·100 mL^−1^
Total coliforms	*N*_0_ = 1100 CFU·100 mL^−1^

**FIGURE 1:**
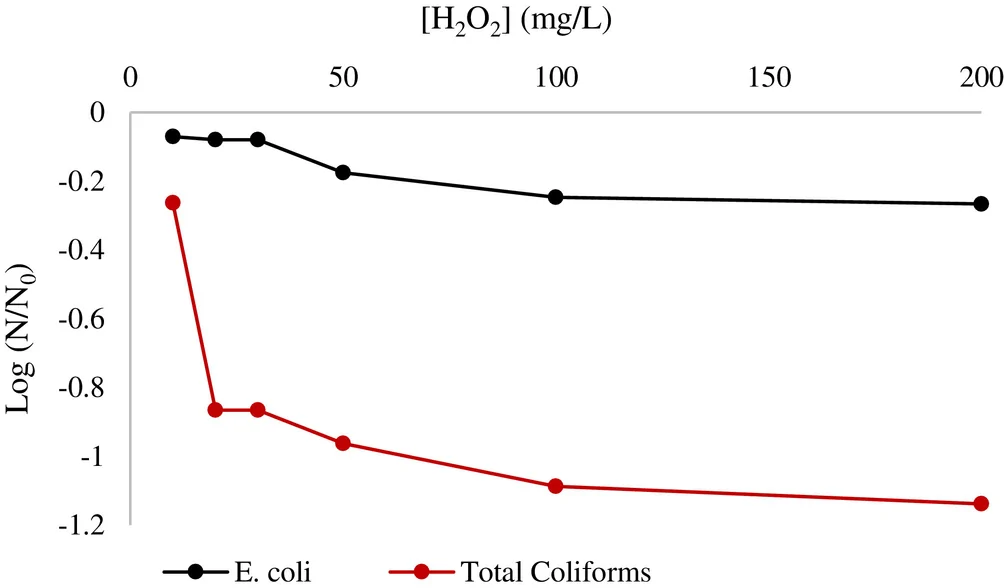
Results of disinfection with hydrogen peroxide at concentrations of 50, 100, 150, and 200 mg/L.

**FIGURE 2:**
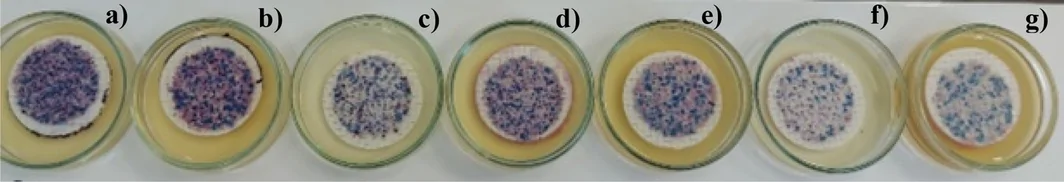
Result of microbiological analysis of river water (a) raw sample; (b) disinfected with 10 mg/L of H_2_O_2_; (c) disinfected with 20 mg/L of H_2_O_2_; (d) disinfected with 30 mg/L of H_2_O_2_; (e) disinfected with 50 mg/L of H_2_O_2_; (f) disinfected with 100 mg/L of H_2_O_2_, and (g) disinfected with 200 mg/L of H_2_O_2_. *Source*: Author 2023.

The results of the disinfection test at different H_2_O_2_ concentrations are shown in Figure 1, with the efficiency of the process represented by Log *N*/*N*_0_. Colony formation for each dosage can be observed in Figure 2.

### 3.2 Acute Effect of Hydrogen Peroxide on *G. tigrina*

The estimated LC_50_ for planarians exposed to hydrogen peroxide for 48 h was 123.55 mg/L (95% confidence interval = 64.71–82.25 mg/L, *R* ^2^ = 0.6). The *R* ^2^ value indicates a moderate level of correlation between concentration and mortality. At lower initial concentrations, no mortality or morphological changes in the organisms were observed. However, at higher concentrations, significant body deformations, where the body was partially or completely degraded, including tail bifurcation, were observed, ultimately leading to the complete disintegration of the organisms. These results highlight the critical thresholds of hydrogen peroxide toxicity and underline the importance of controlling its concentration in aquatic environments to prevent ecological damage.

### 3.3 Effects of Hydrogen Peroxide Exposure on *G. tigrina* Locomotion

**FIGURE 3:**
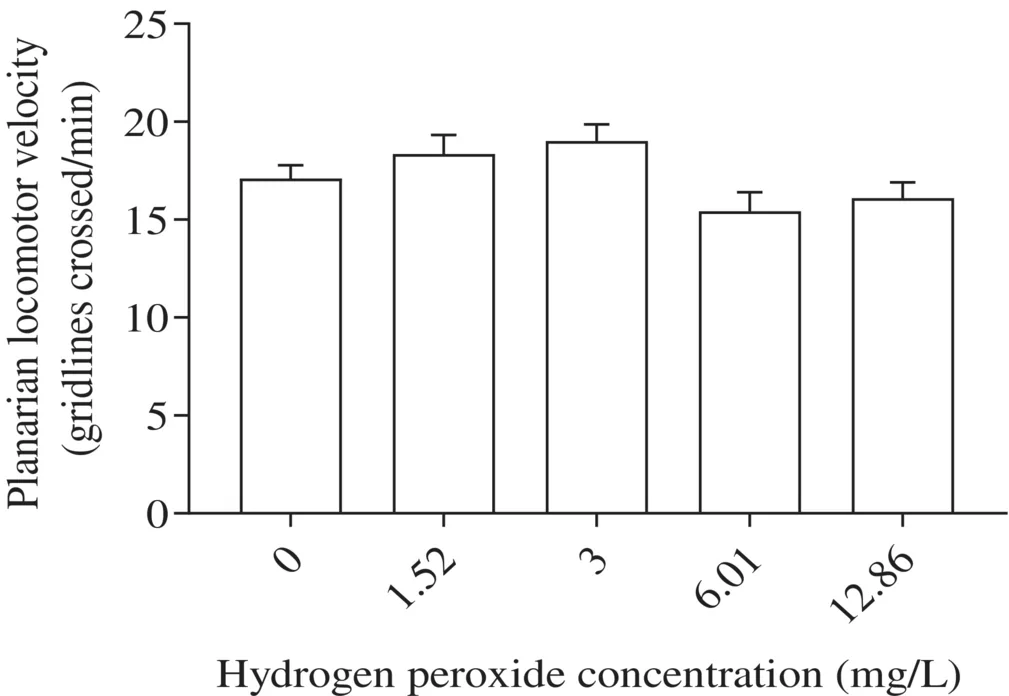
Postexposure locomotor velocity of *Gigrina tigrina* was measured after 8 days of pre‐exposure to 1.52, 3, 6.01, and 12.86 mg/L of hydrogen peroxide, as well as a control (ASTM hard water only). Locomotor velocity is expressed as the number of gridlines crossed and recrossed per minute. Values represent the mean ± SEM (standard error of the mean), *n* = 12. This measurement provides insight into the sublethal effects of hydrogen peroxide on planarian movement, an important indicator of organism health and environmental stress.

The locomotor velocity of planarians (pLMV) was not significantly altered in planarians exposed to hydrogen peroxide compared to the control treatment (F (4,55) = 2.903, *p* = 0.0299) (Figure 3). This suggests that hydrogen peroxide, at the tested concentrations, does not have a significant impact on the locomotion of planarians, indicating the resilience of this behavior to oxidative stress under the experimental conditions.

### 3.4 Effects of Hydrogen Peroxide Exposure on *G. tigrina* Regeneration

**FIGURE 4:**
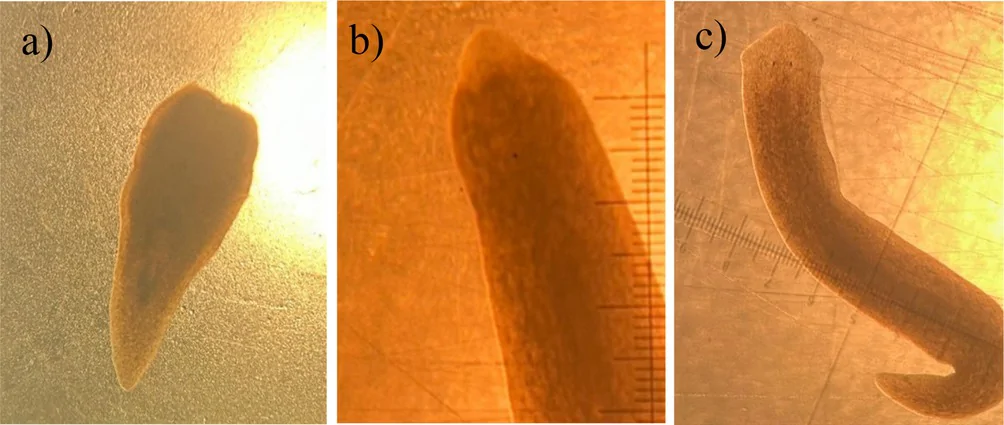
Evaluation of regeneration in *Girardia tigrina* . (a) Organism after decapitation. (b) Measurement of the regenerative blastema. (c) Observation of the emergence of auricles and photoreceptors. *Source*: Author 2023.

**FIGURE 5:**
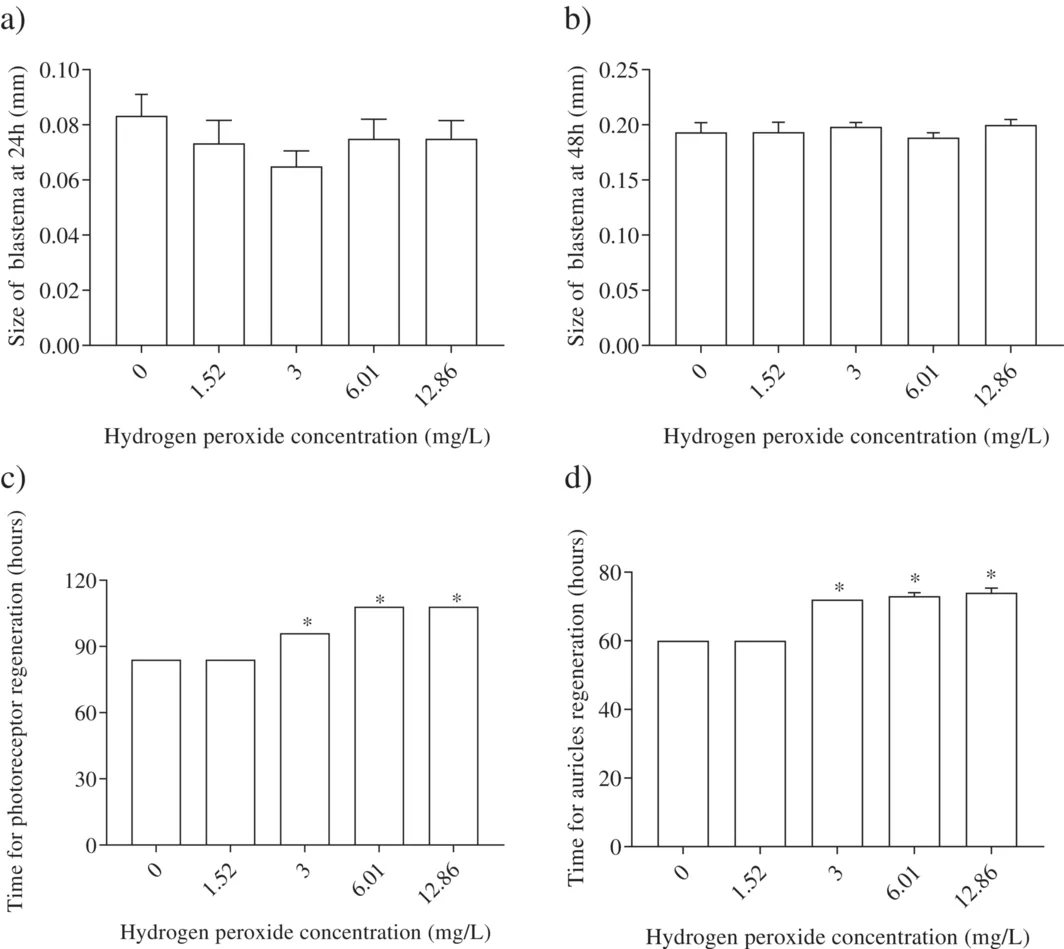
Postexposure size of the blastema (mm) after 24 (a) and 48 h (b), as well as the time to regeneration of photoreceptors (c) and auricles (d) in *Girardia tigrina* decapitated after 8 days of exposure to 1.52, 3, 6.01, and 12.86 mg/L of hydrogen peroxide, compared to a control (ASTM hard water only). Values represent mean ± SEM (standard error of the mean), *n* = 12. *Denotes a significant difference compared to control (Dunnett's test, *p* < 0.05). This analysis highlights the sensitivity of sensory structures to oxidative stress, providing insight into the sublethal effects of hydrogen peroxide on planarian regeneration.

The blastema regeneration process in planarians exposed to hydrogen peroxide, as shown in Figure 4a,b, did not show significant changes compared to the control treatment after 24 h (*F* (4,55) = 0.844, *p* = 0.5034) and 48 h (*F* (4,55) = 0.4959, *p* = 0.7388) shown in Figure 5a,b, respectively. However, the regeneration of photoreceptors (*H* = 59, degrees of freedom [df] = 5, *p* < 0.0001) and auricles (*H* = 55.43, df = 5, *p* < 0.0001) was significantly affected in planarians exposed to hydrogen peroxide shown in Figure 4c, at concentrations above 3 mg/L shown in the graphs of Figure 5c,d, respectively.

These results indicate that while general blastemal regeneration is not impacted, specific sensory structures such as photoreceptors and auricles are highly sensitive to oxidative stress caused by hydrogen peroxide.

### 3.5 Effect of Hydrogen Peroxide Exposure on *G. tigrina* Reproduction

**FIGURE 6:**
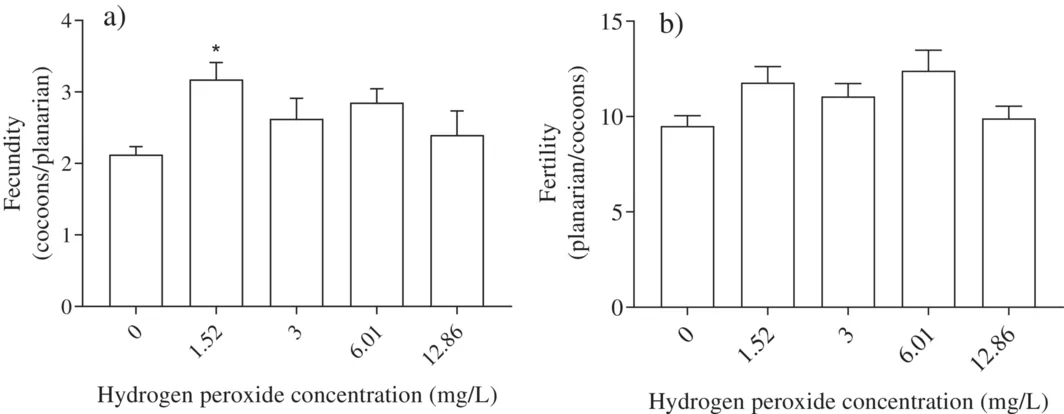
Postexposure reproduction of *Girardia tigrina* planarians after 8 days of pre‐exposure to 1.52, 3, 6.01, and 12.86 mg/L of hydrogen peroxide, and a control (ASTM hard water only). (a) Fecundity of *G. tigrina* , measured as the number of eggs per planarian, after 4 weeks of exposure. (b) Fertility of *G. tigrina* , expressed as the number of planarians per cocoon. Values represent the mean ± SEM (standard error of the mean), *n* = 10. *Denotes a significant difference compared to control (Dunnett's test, *p* < 0.05). These metrics provide insight into the reproductive health of planarians and the potential sublethal effects of hydrogen peroxide exposure.

The fecundity of planarians exposed to hydrogen peroxide (*F* (4,15) = 2.728, *p* = 0.0690) was significantly affected only at the lowest concentration of 1.52 mg/L (Figure 6a). In contrast, no significant difference was found in the fertility of the planarians after exposure to hydrogen peroxide (*F* (4,15) = 2.458, *p* = 0.0905). However, when evaluating the planarians that hatched from the cocoons, morphological abnormalities were observed at the highest concentration (12.86 mg/L of hydrogen peroxide), suggesting potential mutations (Figure 6b). These findings highlight the differential impact of hydrogen peroxide on reproductive metrics and underline the importance of monitoring both fecundity and morphological integrity in ecotoxicological assessments.

## 4 Discussion

The acute effect (LC_50_ 123.55 mg/L) shows that planarians were less susceptible to peroxide exposure than other aquatic organisms, such as the zooplankton *Daphnia carinata* , which has an LC_50_ of 5.6 mg/L, where for this aquatic species H_2_O_2_ induces oxidative stress and its decomposition can lead to small gas embolisms in the exposed tissue area [24].

For organisms such as *S. costatum* , *Brachionus plicatilis* (crustacean), and *Pimephales promelas* (freshwater fish), the acute toxicity of H_2_O_2_ was examined and the concentration (CL_50_) was 1.38, 2.4 and 16.4 mg/L, respectively, proving that high concentrations of H_2_O_2_ are potentially toxic to different aquatic organisms [25].

When evaluating different concentrations and exposure times, H_2_O_2_ induced a decrease in the perception of zebrafish larvae (up to 69%) and affected the movement of adult zebrafish (average speed, average acceleration, distance travel, and activity time) compared to the control group [26].

The applicability of H_2_O_2_ in controlled concentrations can contribute to the control of phytoplankton with high levels of cyanobacteria in wastewater treatment, protecting human and environmental health. According to Barrington and Ghadouani [27], at the lowest dose of H_2_O_2_, a significant exponential decline in phytoplankton groups was observed at approximately 3.0 × 10^−3^ g H_2_O_2_/μg of chlorophyll‐a of phytoplankton, with cyanobacteria and total phytoplankton exhibiting half‐lives of 2.3 and 4.5 h, respectively.

The European Scientific Committee on Toxicity, Ecotoxicity and the Environment, through the risk assessment of H_2_O_2_ and its environmental effects, concluded that there is much toxicity data, where the derivation of a PNECwater (Predicted No‐Effect Concentration for water) of 10 μg/L is consistent with regard to exposure to phytoplankton and bacterioplankton, apparently the most sensitive organisms in the aquatic environment [28].

Regarding the evaluation of auricle and photoreceptor regeneration tests, a 10% delay was observed from a concentration of 3 mg/L, while a significant effect on fecundity was observed from a concentration of 1.52 mg/L. Auricles are highly vascularized structures with tubular and epithelial cells, containing microtubules linked to receptors in the nervous system. These characteristics may help explain why planarians living in environments contaminated with hydrogen peroxide take longer to regenerate their auricles. This suggests a more efficient and rapid absorption of chemical compounds present in the environment, potentially causing the observed delay in auricle regeneration [29].

Studies on species like *Girardia festae*, such as those by Iannacone and Tejada [30], have shown that chemical products can negatively affect the regeneration process in planarians. The significant difference observed in fecundity can be attributed to the hormetic effect. Hormesis is characterized by a biphasic stimulatory effect where a substance, which is detrimental at high doses, exhibits positive effects at low doses. Typically, hormesis effects range between concentrations 30%–60% above control levels and up to 10 times below the no‐observed‐effect concentration (NOEC) [31].

Sublethal exposure of *G. tigrina* to hydrogen peroxide did not result in significant differences in locomotion, blastema regeneration, and fertility between the concentrations evaluated and the control. The toxicity of hydrogen peroxide can be explained by its ability to interact with lipid membranes and other microorganism cell structures, leading to oxidation [32]. Hydrogen peroxide degrades rapidly in light and in the absence of other organic substances, as suggested by Oliveira et al. [33]. This may explain the lack of treatment effects on locomotion and blastema regeneration.

The fecundity of *G. tigrina* did not vary across different concentrations, indicating that hydrogen peroxide degrades quickly and affects primarily the most vascularized and directly contactable structures. Although visually apparent, anomalous regeneration in *G. tigrina* was mainly observed in the syringe part, likely attributed to chemical stress in the environment; however, even planarians displaying deformities retained their ability to regenerate.

Thus, because planaria are used as bioindicators, changes in both physiology and morphology pose a risk to aquatic ecosystems due to toxicity to nontarget organisms [4].

**FIGURE 7:**
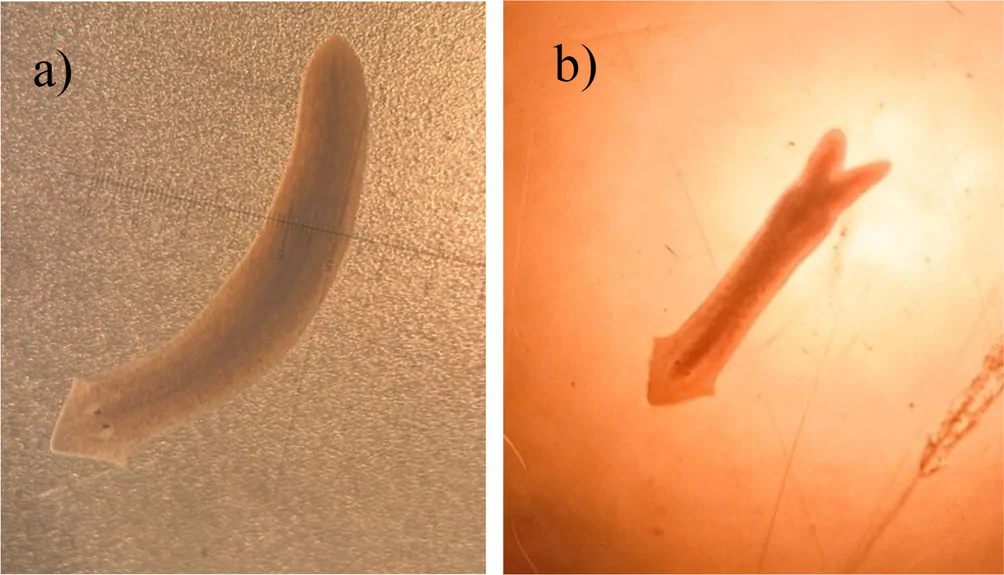
Morphological comparison of *Girardia tigrina* . (a) Control organism, without structural alterations. (b) Representation of abnormality formed after exposure to concentration of 12.86 mg/L of H_2_O_2_. *Source:* Author 2023.

However, when evaluating planaria that hatched from the cocoons, an abnormality was noted at the highest concentration, where two planaria showed morphological changes. In these specimens, forked tails were observed (Figure 7b), while in the control group, organisms showed no alterations (Figure 7a).

The presence of hydrogen peroxide in an organism can induce oxidative stress in the aquatic environment and lead to the destruction of the stable oxidation–reduction state, the degradation of cell membranes of other organisms, and the degradation of biological macromolecules [26].

Considering the various environmental, natural, and anthropogenic factors that can contribute to its formation, decomposition, and concentration, the Japanese Ministry of the Environment included H_2_O_2_ in the list of “Priority Chemicals” in its chemical control legislation, through the monitoring of 12 rivers over a 3‐year period [25].

Regarding genotoxicity, data from the UK Health Security Agency indicate that hydrogen peroxide has mutagenic potential in vitro systems; however, it is not possible to conclude that hydrogen peroxide is mutagenic in vivo [34]. This is the first in vivo study to report the appearance of morphological abnormalities in planarians from exposure to H_2_O_2_.

## 5 Conclusion

The study findings demonstrated significant impacts on planarian fecundity, regeneration, and morphology, including the development of bifurcated tails. These results clearly illustrate that while hydrogen peroxide serves to purify water, it also adversely affects nontarget organisms such as planarians, which play a critical role in aquatic ecosystem health.

The development of a forked tail in planarians signifies a substantial physiological change that could threaten their survival and reproductive capacity. These findings underscore the importance of considering aquatic biodiversity when selecting water disinfection agents like hydrogen peroxide or similar compounds for use in natural settings.

Therefore, this study emphasizes the necessity for a cautious and informed approach in choosing water disinfection agents. It is crucial to evaluate not only their effectiveness in pathogen elimination but also their potential negative impacts on aquatic life. This research contributes to our understanding of the risks associated with hydrogen peroxide and provides essential data for the conservation of aquatic life and sustainable water resource management.

## Author Contributions

**Letícia Queiroz Almeida:** investigation, writing – original draft preparation. **Thayrine Dias Carlos:** methodology, investigation. **Leydiane Barbosa Bezerra:** investigation, writing – review and editing. **Rone S. Barbosa:** investigation, data curation, writing – review and editing. **Nelson Luís Gonçalves Dias Souza:** writing – review and editing, supervision, funding acquisition. **Douglas Henrique Pereira:** writing – review and editing, supervision, funding acquisition. **Renato Almeida Sarmento:** conceptualization; methodology; writing – review and editing; supervision; funding acquisition. **Grasiele Soares Cavallini:** conceptualization, methodology, writing – review and editing, supervision, funding acquisition.

## Funding

This work was supported by the (Coordinação de Aperfeiçoamento de Pessoal de Nível Superior [CAPES‐BRAZIL]) under Grant Financing Code 001 CAPES, PDPG‐POSDOC (Programa de Desenvolvimento da Pós‐Graduação [PDPG] Pós‐Doutorado Estratégico—Postgraduate Development Program [PGDP] Strategic Postdoctoral) under Grant No. 88887.798251/2022‐00; the PROPESQ and the Federal University of Tocantins for financial assistance; Conselho Nacional de Desenvolvimento Científico e Tecnológico (CNPq‐Brazil) Grant No. 305595/2024‐5, 443317/2024‐0; and Tocantins Research Support Foundation (FAPT).

## Ethics Statement

The authors have nothing to report.

## Consent

The authors have nothing to report.

## Conflicts of Interest

The authors declare no conflicts of interest.

## Data Availability

The data that support the findings of this study are available from the corresponding author upon reasonable request.
